# Pea aphid *Acyrthosiphon pisum* sequesters plant-derived secondary metabolite L-DOPA for wound healing and UVA resistance

**DOI:** 10.1038/srep23618

**Published:** 2016-03-23

**Authors:** Yi Zhang, Xing-Xing Wang, Zhan-Feng Zhang, Nan Chen, Jing-Yun Zhu, Hong-Gang Tian, Yong-Liang Fan, Tong-Xian Liu

**Affiliations:** 1 State Key Laboratory of Crop Stress Biology for Arid Areas, College of Plant Protection, Northwest A&F University, Yangling, Shaanxi, 712100, China; 2 Key Laboratory of Integrated Pest Management on the Loess Plateau of Ministry of Agriculture, College of Plant Protection, Northwest A&F University, Yangling, Shaanxi, 712100, China

## Abstract

Herbivores can ingest and store plant-synthesized toxic compounds in their bodies, and sequester those compounds for their own benefits. The broad bean, *Vicia faba* L., contains a high quantity of L-DOPA (L-3,4-dihydroxyphenylalanine), which is toxic to many insects. However, the pea aphid, *Acyrthosiphon pisum*, can feed on *V. faba* normally, whereas many other aphid species could not. In this study, we investigated how *A. pisum* utilizes plant-derived L-DOPA for their own benefit. L-DOPA concentrations in *V. faba* and *A. pisum* were analyzed to prove L-DOPA sequestration. L-DOPA toxicity was bioassayed using an artificial diet containing high concentrations of L-DOPA. We found that *A. pisum* could effectively adapt and store L-DOPA, transmit it from one generation to the next. We also found that L-DOPA sequestration verity differed in different morphs of *A. pisum*. After analyzing the melanization efficiency in wounds, mortality and deformity of the aphids at different concentrations of L-DOPA under ultraviolet radiation (UVA 365.0 nm for 30 min), we found that *A. pisum* could enhance L-DOPA assimilation for wound healing and UVA-radiation protection. Therefore, we conclude that *A. pisum* could acquire L-DOPA and use it to prevent UVA damage. This study reveals a successful co-evolution between *A. pisum* and *V. faba.*

Aphids feed on the fluid in plant phloem for their survival. The high reproductive capacity makes many aphid species became serious agricultural pests in the world^1^. Aphids vary in their host range from oligophagous to polyphagous. The pea aphid, *Acyrthosiphon pisum* (Harris), is specialized on only some species of legumes, and is considered as an oligophagous specie^2^; whereas some species, such as the green peach aphid, *Myzus persicae* (Sulzer), feeding on over 400 plant species in nearly 50 plant families, is a polyphagous species^3^.

Host plants of aphids display many defenses from genetic modifications to chemical changes, which could be directly harmful for aphids or attract natural enemies^4^. Meanwhile, aphids develop various adaptations such as using immune system to manage secondary metabolites and against its natural enemies^5^,^6^,^7^,^8^,^9^. In this typical tritrophic system, effects of host defense metabolites are normally negatively against aphids and positively benefit to aphid’s natural enemies^4^.

There is also another relationship existing among herbivores, their hosts, and natural enemies. Many herbivorous insects can use plant-synthesised and normally insect-harmful chemicals to benefit themselves by ingesting and storing them in their body tissues or integument, which is known as sequestration to herbivorous pests^10^. More than 250 insect species in at least 6 orders have been recorded to sequester their hosts’ metabolites from at least 40 plant families^10^. For example, it has been found that pyrrolizidine alkaloids in leguminous plants can be acquired by a lepidopteran, *Utetheisa ornatrix* (L.), and then be carried to the moth’s eggs to protect them from the damage of an egg parasitoid, *Trichogramma ostriniae* Pang et Chen, and a green lacewing, *Ceraeochrysa cubana* (Hagen)^11^,^12^,^13^. This host adaptation of insects is usually specific and observed in oligophagous species. The key chemicals in this relationship are alkaloids or some complex compounds such as phenolic glycosides, cyanogenic glycosides and glucosinolates, and most of these chemicals are detected in relatively high amount in both host plants and herbivorous that consuming these plants^10^.

The broad bean, *Vicia faba* L., a host plant of *A. pisum* and other aphid species, has already been proved containing high quantity of L-DOPA (L-3,4-dihydroxyphenylalanine)^14^,^15^, which is a non-protein amino acid involving in many metabolizing of both animals and plants. In animals, L-DOPA is the precursor of the neurotransmitters dopamine, norepinephrine, and epinephrine, which are collectively known as catecholamines. Many behaviuors of animals are modified by these neurotransmitters^16^,^17^,^18^. L-DOPA is also a key chemical involving in sclerotization and melanization of insects, influencing insect’s development and immunity^9^,^19^,^20^. In pharmacology, L-DOPA is used to treat Parkinson’s disease, and the natural resource of the L-DOPA is present in legumes^15^. L-DOPA is biosynthesised via the shikimate pathway in plants as a secondary metabolite, the precursor of some alkaloids in several plants, like mescaline, berberine, morphine, heroin, and papaverine; and most of them can negatively and directly affect animal nervous system^21^,^22^,^23^.

Legume plants contain substantial amounts of L-DOPA in the leaves and bean pods, such as *V. faba*, the velvet bean, *Mucuna pruriens*, and the seeds of *Cassia* and *Bauhinia* trees, making them the most valuable commercial plants for the treatment of Parkinson’s disease^14^,^15^,^22^,^24^. The functions of L-DOPA in these plants on insects have still not been fully understood. Recent studies have shown L-DOPA as an important secondary metabolite for chemical defense against herbivores in plants^25^. Previous studies on a variety of bruchid species to the honey locust, *Gleditsia triacanthos*, seeds have shown the toxicity of L-DOPA^26^,^27^. Other evidence suggested the L-DOPA pathway acts as an anti-herbivore defense, reducing consumption by snails and isopods in the marine green alga, *Ulvaria obscura*^28^. Studies on the grain aphid, *Sitobion avenae* (Fabricius), indicated that the concentration of L-DOPA in wheat had a negative correlation to the intrinsic rate of natural increase r_m_^29^. In addition, it has been found that L-DOPA pathways in plants might play a role in response to stress conditions such as infection by bacteria, which might be involved in many aspects of development and growth of plants, and might also modify carbohydrate metabolism in plants^30^.

Although L-DOPA is harmful to some herbivorous pest insects, it is also a key chemical involved in animal immune system^9^. For instance, aphids need to challenge natural enemies, including entomopathogens, parasitoids and predators, and defense all of chemicals in host plants. These challenges have caused aphids to evolve numerous L-DOPA related defense mechanisms, such as clotting, encapsulation, phagocytosis and melanization, as well as initiate immune pathways for defense. Generally, L-DOPA was converted from tyrosine by tyrosine hydroxylase (tyrosine 3-monooxygenase, TH); it is a rate limiting step in synthesis of L-DOPA and dopamine in animals; and it could be inhibited by enzyme α-methyltyrosine (metirosine)^5^,^6^,^7^,^9^,^31^,^32^. Cellular encapsulation is a defense response against large foreign objects such as parasitoid eggs or bacteria that cannot be phagocytized by haemocytes, and melanization is also involved in this process, and L-DOPA is a key chemical involved^7^,^31^. The melanization reaction provides toxic chemicals like dopaquinone, which is also involved in encapsulation on the cuticle with cuticular proteins^19^,^20^,^33^. These reactions can cause capsules and display dark color on insect cuticle. Phenoloxidases (POs) are thought to be key enzymes in melanin synthesis which can hydroxylate monophenols and oxidise odiphenols to quinones, and prophenoloxidase (proPO) is a modified form of the complement response found in some invertebrates. ProPO1 and proPO2 were found and studied in *A. pisum* in previous work^9^,^33^. Researches in human and other animals also showed the important relationship between melanization and ultraviolet (UVA) radiation-protection, and melanics always are highly resistant to UVA radiation^34^,^35^,^36^,^37^. On the other hand, UVA irradiation can cause melanin pigment reorganization and generation in animal tissues to protect from damage of inner DNA^38^,^39^,^40^.

Considering the dramatically high amount of L-DOPA in broad bean with aphids on it, amino acid-compound-like L-DOPA has not been recorded in any insect sequestration. We hypothesized that L-DOPA might be stored and used by aphids in their metabolic system. Therefore, we attempted to investigate whether *A. pisum* could sequester L-DOPA from *V. faba,* and to determine how *A. pisum* uses this chemical to benefit itself. In this study, we analyzed the concentrations of L-DOPA and dopamine in two groups of *A. pisum* and their two host plants (the broad bean *V. faba* and the white clover *Trifolium repens*), and verified related genes in this pathway; and we then compared melanization and UVA protection between these two *A. pisum* populations fed on the two host plants.

## Results

### L-DOPA contents in *A. pisum* and two host plants

**Figure 1:**
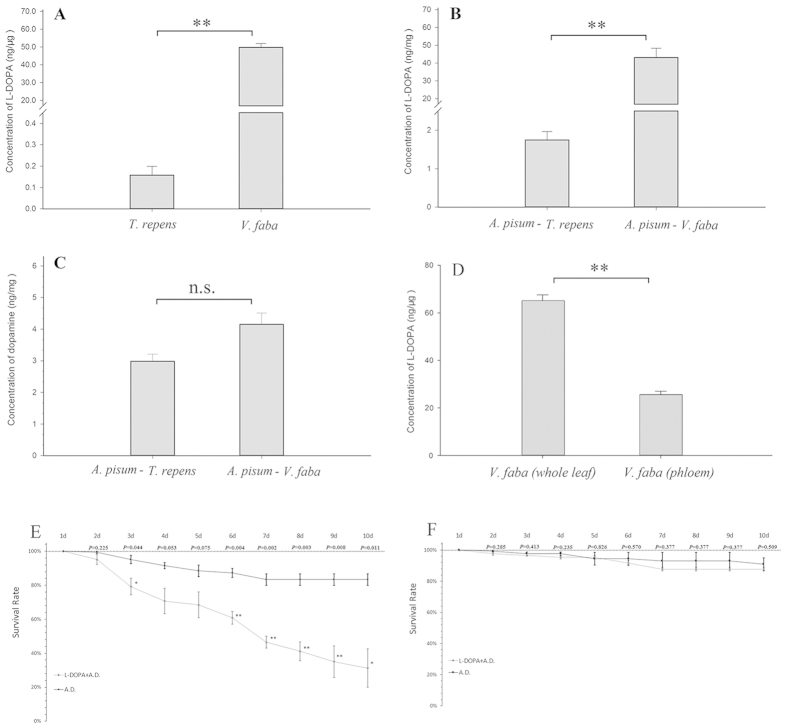
L-DOPA contents in *Vicia faba* and *Trifolium repens* (**A**) and L-DOPA (**B**) dopamine (**C**) contents in *Acyrthosiphon pisum* reared separately on *V. faba* and *T. repens*; L-DOPA concentrations in phloem of *V. faba* were detected and shown in (**D**) L-DOPA amounts in leaves of *V. faba* were extracted by grinding as a positive control; survival rate of aphids treated with L-DOPA (20 mM) by feeding artificial diets. Survival rate of *A. pisim* fed on *T. repens* (n_AD_ = 150, n_DOPA_ = 150) was shown in (**E**) Survival rate of *A. pisim* fed on *V. faba* (n_AD_ = 150, n_DOPA_ = 150) was shown in (**F**).

To understand the L-DOPA contents differences among our experimental plants, LC/MS results showed that the L-DOPA concentration in *V. faba* was more than 300 fold higher (49.750 ng/μg) than that in *T. repens* (0.157 ng/μg) (*t* = −22.571, df = 22, *P* < 0.0001) (Fig. 1A), which was also significantly higher (*F* = 102.607, df = 4, 38, *P* < 0.0001) than that in *Capsicum annuum* (0.184 ng/μg),*Nicotiana tabacum* (0.026 ng/μg), and *Triticum aestivum* (0.014 ng/μg) (Fig. S2A). The amount of L-DOPA in *V. faba* could reach 5% of total dry weight of the plant (Fig. 1A). EDTA extraction experiments showed about half amounts of L-DOPA (*t* = −13.791, df = 8, *P* < 0.0001, (Fig. 1D) were located in phloem which could be fed by aphid, and no L-DOPA was detected in phloem of *T. repens, C. annuum, N. tabacum* and *T. aestivum* (Fig. S2D).

The contents of L-DOPA in the two groups of *A. pisum* were significantly different, and the concentration of L-DOPA in the aphids fed on *V. faba* (43.170 ng/mg) was 25-fold higher than that *A. pisum* fed on *T. repens* (1.745 ng/mg) (*t* = −7.959, df = 7, *P* < 0.0001) (Fig. 1B). Dopamine concentrations in the two aphid groups were not significant different (2.982 and 4.151 ng/mg, respectively) (*t* = −2.000, df = 26, *P* = 0.056) (Fig. 1C).

The contents of L-DOPA and dopamine from three strains of *M. persicae,* a *V. faba* adapted-strain, a *C. annuum* adapted strain, and a *N. tabacum* adapted strain, and *S. avenae* and the bird cherry-oat aphid, *Rhopalosiphum padi* L., reared on *T. aestivum* were not significantly different (*F* = 72.968, df = 6, 38, *P* < 0.0001). Although the highest concentration of L-DOPA was found in *A. pisum* from *V. faba*, the amounts of dopamine were not significantly different among the aphids in all the other host plants (*F* = 2.301, df = 6, 42, *P* = 0.052) (Fig. S2B,C).

### Toxicity of L-DOPA to Aphids

In L-DOPA toxicity analysis, *A. pisum* from *V. faba* had a 30% higher survival rate (>85%) (Fig. 1F) compared with that from *T. repens* (Fig. 1E). The aphids fed on an artificial diet without L-DOPA had a similar survival rate to that fed on the artificial diet with L-DOPA. The results of other experimental aphids showed that only *M. persicae* from *V. faba* could survive at a relatively low rate on the artificial diet containing L-DOPA while all other aphids could not (Fig. S3).

### Concentrations of L-DOPA in different morphs and tissues of *A. pisum*

**Figure 2:**
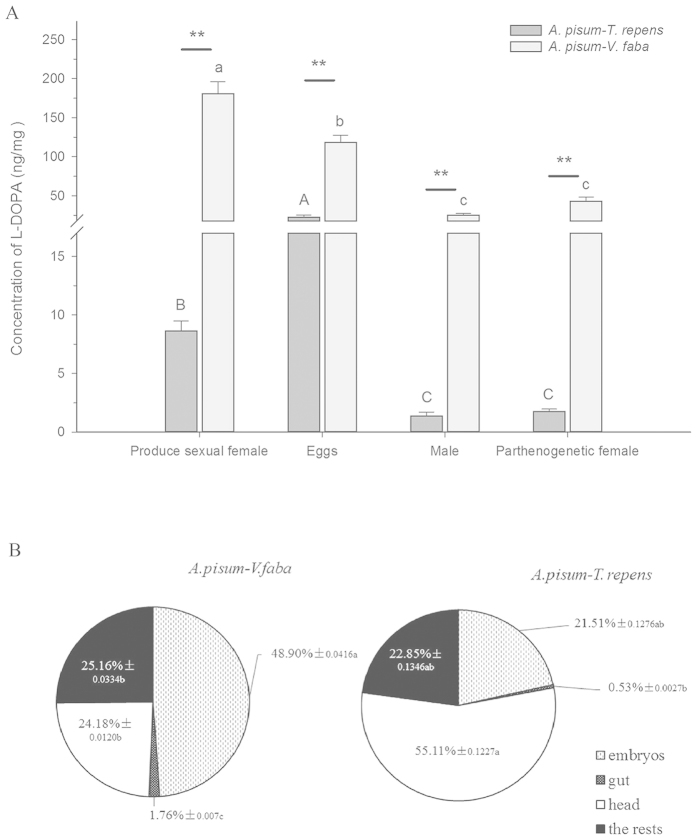
L-DOPA concentrations in different morphs of *Acyrthosiphon pisum* fed on two hosts, *Vicia faba* and *Trifolium repens*. (**A**) L-DOPA concentrations; (**B**) Proportion of L-DOPA from each body part. Each value represents the mean ± SEM.

The L-DOPA concentrations in different aphid morphs showed significant differences (parthenogenetic female: *t* = 7.963, df = 7, *P* < 0.0001; sexual producing female: *t* = 11.425, df = 4, *P* < 0.0001; male: *t* = 7.310, df = 8, *P* < 0.0001; egg: *t* = 11.315, df = 4, *P* < 0.0001) (Fig. 2A). However, both male and parthenogenetic female contained relatively lower amounts of L-DOPA than the sexual female and eggs. The amounts of L-DOPA were significantly different among the aphid morphs (*A. pisum* from *V. faba: F* = 66.091, df = 3, 17, *P* < 0.0001*, A. pisum* from *T. repens: F* = 77.646, df = 3, 11, *P* < 0.0001). However, the eggs of the *A. pisum* from *T. repens* had the highest level of L-DOPA among the four aphid morphs.

Different tissues of the two *A. pisum* populations contained different amounts of L-DOPA (*A. pisum* from *V. faba: F* = 48.812, df = 3, 8, *P* < 0.0001*, A. pisum* from *T. repens: F* = 4.104, df = 3, 8, *P* = 0.049) (Fig. 2B). L-DOPA was mostly located in the head and thorax (55.11%). The distribution of L-DOPA in the *A. pisum* from *V. faba* was different from that of *T. repens*, and the embryos (48.90%) contained the highest amount of L-DOPA among all tissues.

### Effect of L-DOPA on the L-DOPA self-biosynthesis

**Figure 3:**
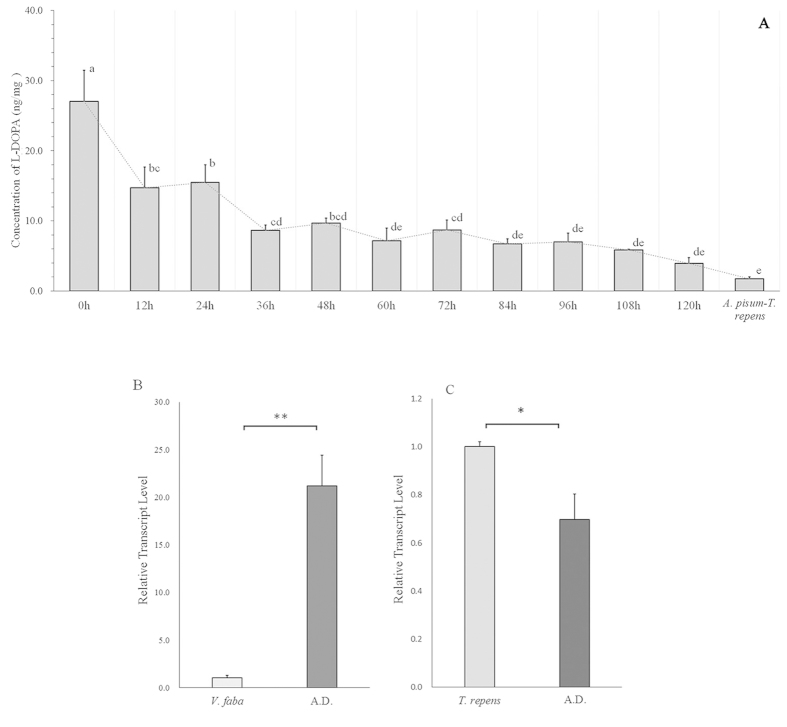
(**A**) Declining trend of L-DOPA concentrations after *Acyrthosiphon pisum* on *Vicia faba* were transferred into an artificial diet, and the last bar represents the L-DOPA concentrations in *A. pisum* fed on *Trifolium Repens.* (**B**) Relative transcript levels of *TH* from the aphids were reared on *V. faba* and the artificial diet; and (**C**) Relative transcript levels of *TH* from the aphids were reared on *T. repens* and the artificial diet. Each value represents the mean ± SEM.

L-DOPA concentration declined continuously after the aphids collected from *V. faba* had been transferred to the artificial diets (*F* = 13.910, df = 11, 28, *P* < 0.0001), but the concentration of L-DOPA in the transferred *A. pisum* was always higher than that in the aphids fed on *T. repens* than those fed on *V. faba* (Fig. 3A).

The endogenous production of L-DOPA in the aphids could be disrupted without L-DOPA assimilation. The transcription analysis showed that *TH* expression (up-regulation) in AD treatment was 20-fold higher than in the control (the aphids reared on *V. faba*) (*t* = −6.201, df = 4, *P* = 0.003) (Fig. 3B). On the contrary, *TH* expression in the *A. pisum* from *T. repens* was about 30% down-regulated in AD compared with that in *T. repens* (*t* = 0.048, df = 4, *P* = 0.048) (Fig. 3C).

### Wound healing

**Figure 4:**
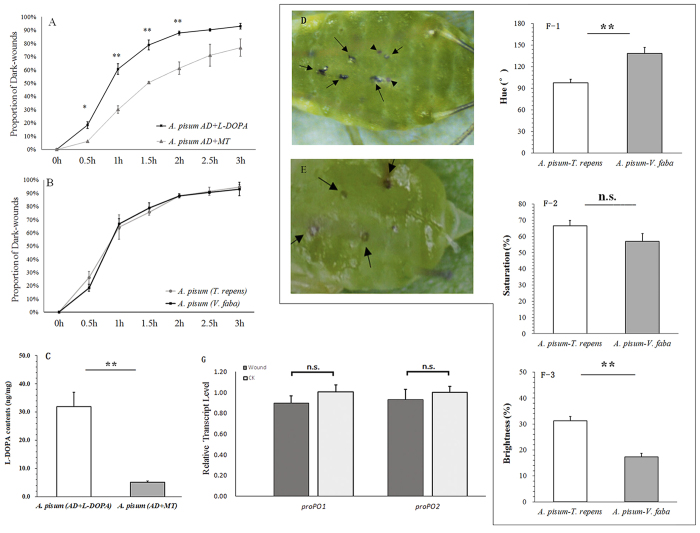
(**A**) Difference in melanization efficiency of *Acyrthosiphon pisum* treated with L-DOPA and α- methyltyrosine; (**B**) Difference in melanization efficiency of *A. pisum* from *Vicia faba* and *Trifolium repens;* (**C**) L-DOPA contents from the aphids collected from two diets. Each value represents the mean ± SEM from independent determinations. (**D**) Wounds (pricking by glass needle) in melanization (arrows); (**E**) Eyes of embryos (arrow heads); (**F**) Color information (base on HSB, hue, saturation and brightness) in wound areas; and (**G**) Relative transcript level of *proPO1* and *proPO2* after treated by glass needle. Each value represents the mean ± SEM from independent determinations.

In wound healing experiment with artificial diet, the aphids treated with α-methyltyrosine (*A. pisum* MT) had a lower proportion of melanized wounds than those fed with L-DOPA (0.5 h: *P* = 0.039, 1 h: *P* = 0.003; 1.5 h: *P* = 0.002; 2 h: *P* = 0.005) (Fig. 4A). In wound healing experiment with different hosts, The proportions of melanized wounds in the aphids fed on *V. faba* and *T. repens* were similar (Fig. 4B), although the amounts of L-DOPA were different (*t* = 5.145, df = 5, *P* < 0.0001) (Fig. 4C). The melanin in the aphids fed on *V. faba* was slightly darker than that in the aphids fed on *T. repens* in the first few hours (Fig. 4D–F). Cuticular color analysis showed significant differences in hue (*t* = −4.072, df = 34, *P* < 0.0001) and brightness (*t* = 6.524, df = 42, *P* < 0.0001) 1 hour after treatment, but not in saturation (*t* = 1.633, df = 34, *P* = 0.111) (Fig. 4F).

The expression of melanization related genes (*proPO1* and *proPO2*) were shown in Fig. 4G. The transcriptional levels of *proPO1* and *proPO2* in the wounded aphids were not different from the aphids in the control (*proPO1*: *t* = −0.196, df = 4, *P* = 0.335; and *proPO2*: *t* = −0.639, df = 4, *P* = 0.557) (Fig. 4G).

### UVA Resistance

**Figure 5:**
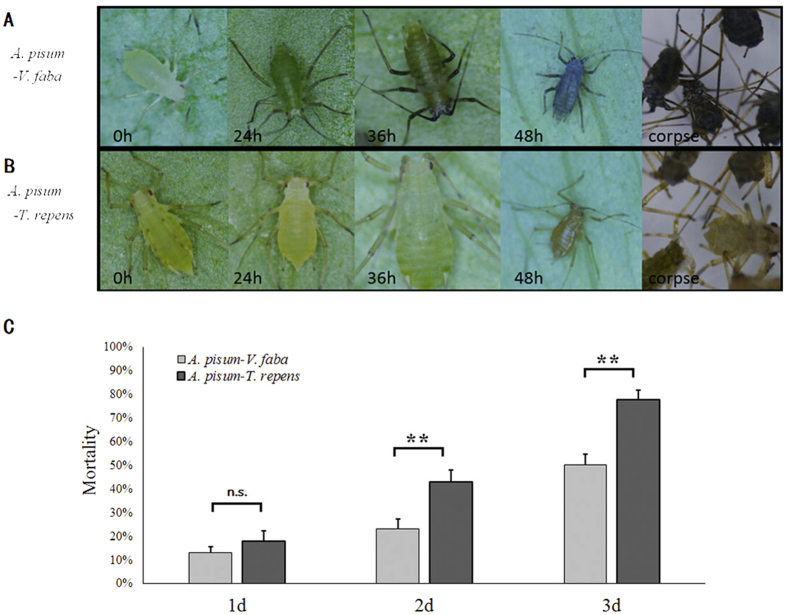
Melanization level in 48 hours difference of *Acyrthosiphon pisum* after 365 nm Ultraviolet radiation (UVA radiation), and (**A**) for *Vicia faba,* and (**B**) for *Trifolium repens*. (**C**) Mortality was assessed 3 days after UVA radiation. Each value represents the mean ± SEM from independent determinations.

In UVA-resistances experiments. *A. pisum* from *V. faba* showed greater melanization (darker) (Fig. 5A) than that of *A. pisum* fed on *T. repens* (Fig. 5B). UVA treatments did not cause significant mortality at the beginning of treatment for the aphids that fed on the two different host plants. However, the mortality of *A. pisum* fed on *T. repens* was greater than those fed on *V. faba* 2 days after treatment (day1: *t* = 1.056; df = 6; *P* = 0.331; day 2: *t* = 2.826; df = 18; *P* = 0.009; day 3: *t* = 4.727; df = 7; *P* = 0.002) (Fig. 5C).

**Figure 6:**
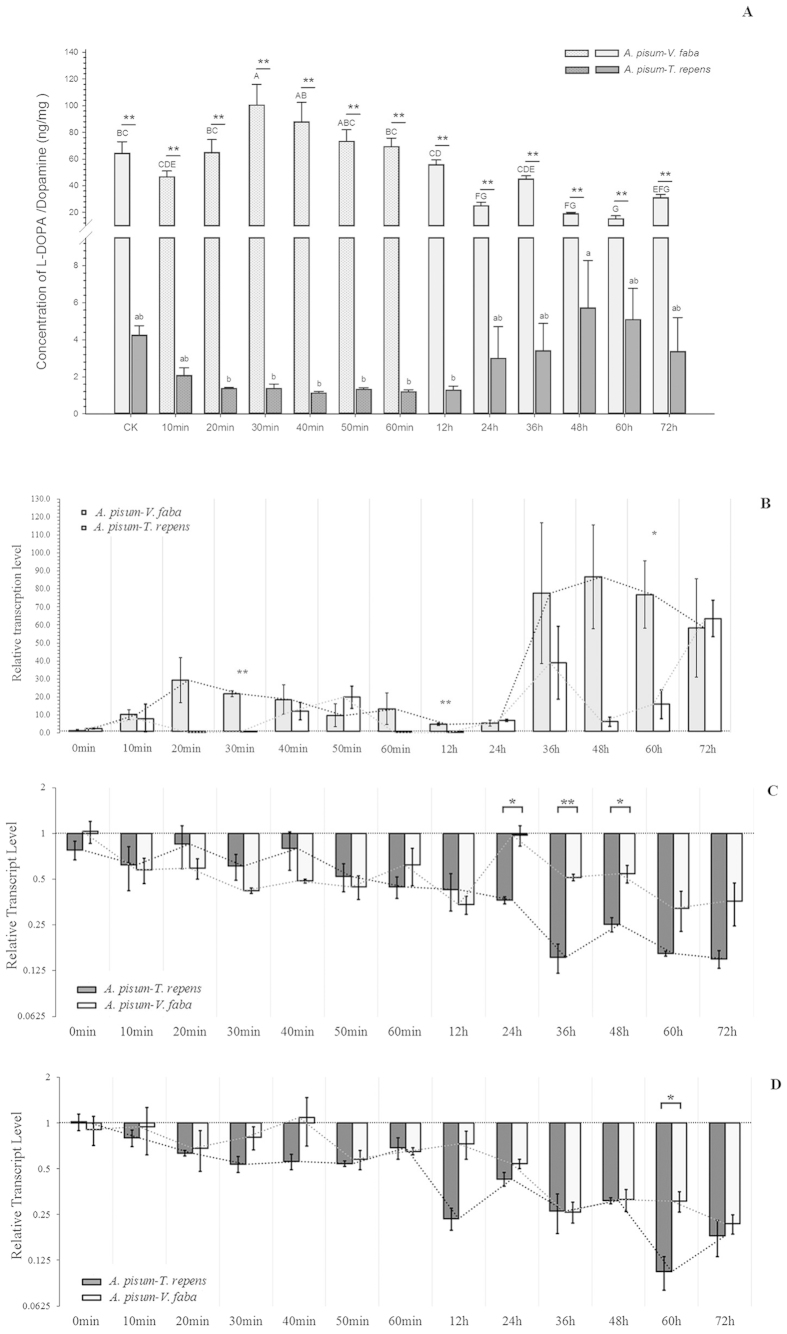
(**A**) Dynamic L-DOAP concentration during UVA radiation and 72 hours after treatment; (**B**) Relative transcript levels of *TH* after UVA radiation treatment to *Acyrthosiphon pisum* from two hosts in 72 hours; (**C**) Relative transcript levels of *proPO1;* and (**D**) *proPO2* after UVA radiation treatment to *A. pisum* from two hosts in 72 hours. Each value represents the mean ± SEM.

The effects on L-DOPA concentrations after UVA radiation on the aphids fed on *V. faba* and *T. repens* differed dramatically (Fig. 6A). The aphids fed on *V. faba* contained more L-DOPA than those fed on *T. repens*. L-DOPA concentrations of the aphids from *V. faba* increased in the first 20 min, peaked in 30 min after treatment, and slowly declined in the next 3 days. In contrast, the L-DOPA concentrations of the aphids from *T. repens* were low and stabilized for the first 12 h, and then increased in the next 3 days.

The expression levels of *TH* (Fig. 6B)*, proPO1* (Fig. 6C) and *proPO2* (Fig. 6D) also changed over time. *TH* expression was relatively stable in the first day, and changed dramatically thereafter (Fig. 6B). The expressions of *TH* between the aphids from the two host plants varied over time, especially 3 days after treatment. The expressions of *proPO1* and *proPO2* were slowly declining (Fig. 6C,D). The *proPO1* expression levels between the aphids fed on *V. faba* and *T. repens* were similar at the first 12 h, and then the expression level of the aphids fed on *V. faba* became higher than those fed on *T. repens* until 48 h (24 h: *t* = −4.108; df = 4; *P* = 0.015; 36 h: *t* = −8.355; df = 4; *P* = 0.001 and 48 h: *t* = −3.777; df = 4; *P* = 0.019) (Fig. 6C). However, the expression levels of *proPO2* between the aphids fed on the two host plants were not significantly different, although they showed significantly higher in aphids fed on *V. faba* (*t* = 3.742; df = 4; *P* = 0.020) at 60 h (Fig. 6D).

**Figure 7:**
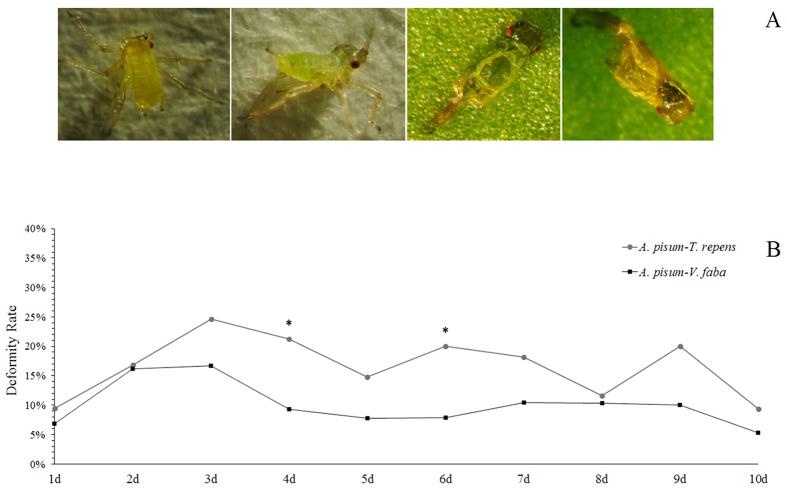
(**A**) Deformity-descendants laid by UV-treated mothers; (**B**) Deformity-descendants rate of *Acyrthosiphon pisum* from two hosts after UVA radiation treatment in 10 days.

UVA treatments caused deformity of aphid offspring (Fig. 7). Generally, the aphids fed on *T. repens* had a higher rate of deformity-descendants than those fed on *V. faba*, but these rates were only significantly different on day 4 (χ^2^ = 4.615, df = 1, *P* = 0.032) and on day 6 (χ^2^ = 4.279, df = 1, *P* = 0.039) (Fig. 7B).

The contents of L-DOPA after the aphid host transfer, UVA and injury treatments are shown in Table 1. The contents of L-DOPA in the *A. pisum* that were originally reared on *V. faba* (high L-DOPA diet) and then were transferred to the same host *(V. faba*) differed significantly among the three treatments (F = 6.011, df = 2, 9, *P* = 0.022); the aphids on the same host had lower amounts of L-DOPA than the UVA-treated aphids, but had a similar amount of L-DOPA to the injured aphids, while the UVA-treated and injured aphids had a similar amount of L-DOPA. The contents of L-DOPA in the *A. pisum* that were originally reared on *V. faba* and then were transferred to *T. repens* (low L-DOPA diet) were not significantly different among the three treatments (F = 0.555, df = 2, 10, P = 0.591). Similarly, the contents of L-DOPA in the *A. pisum* that were originally reared on *T. repens* and then were transferred to *V. faba* were not significantly different among the three treatments (F = 0.080, df = 2, 10, P = 0.923). Again, the contents of L-DOPA in the *A. pisum* that were originally reared on *T. repens* and then were transferred to the same host (*T. repens*) were also not significantly different among the three treatments (F = 0.475, df = 2, 10, P = 0.636).

## Discussion

Our results from the L-DOPA toxicity and concentration experiments confirmed that L-DOPA could be used as a defense chemical for *V. faba* against herbivores, and L-DOPA has been proved to be sequestered by *A. pisum*. The results also showed that L-DOPA could be used by *A. pisum* as defense chemicals. The concentrations of L-DOPA varied in different morphs of *A. pisum*, and the eggs had the highest concentration. L-DOPA can be transferred from one generation to the next, and the newborn nymphs from *A. pisum* reared on *V. faba* had a high amount of L-DOPA. The results further indicated that L-DOPA might be used in wound healing of the aphids, and could enhance UV-irradiation protection for the aphids as indicated by tissue melanization, and L-DOPA assimilation would be enhanced as needed.

There are plenty of resistance mechanisms in legumes against herbivores^41^, and L-DOPA is harmful to some herbivores in some legumes and marine plants^26^,^27^,^28^. Our data proved that L-DOPA might have similar functions in *V. faba.* The mortality of *A. pisum* from *T. repens* showed that this aphid population had lost some of their adaptation and resistance to L-DOPA. A trace amount of L-DOPA was also detected in *C. annuum, N. tabacum* and *T. aestivum*, but these amounts are not high enough to cause any negative effect to herbivores.

Chemical sequestration in herbivores has been previously proved^10^. In our study, L-DOPA concentration (L-DOPA sequestration) in the tested aphids varied, and this chemical sequestration was specific to *A. pisum. A. pisum* fed on *V. faba* contained the highest amount of L-DOPA. We found that in the *A. pisum* fed on *V. faba*, only a small part of L-DOPA was involved in dopamine metabolizing pathway and turned into dopamine for behavior modification or in melanization for cuticle development and immune system, and similar results have been reported in the literature^9^,^16^,^19^,^42^. This phenomenon suggested that *A. pisum* could sequester L-DOPA, and may have a L-DOPA throttle system to avoid extra L-DOPA over-converting into downstream products, and protecting normal physiological activities from being disrupted. In the previous works of chemical sequestration, most of the sequestered chemicals are externally-derived and relatively large molecules such as toxic alkaloids, and these chemicals mostly have specific functions^10^. Because L-DOPA originally exists in aphids and other animals, it might be more compatible to animal metabolic system. L-DOPA, as a non-protein amino acid showing both positive and negative effects, is built by a relatively simple molecule structure, and it could be controlled or modified by adapted-aphids for wide uses.

Based on the preliminary work of L-DOPA and dopamine distribution in *A. pisum* collected from the two hosts (Fig. 2B), we found that the embryos had the most L-DOPA in the *A. pisum* fed on *V. faba*. This evidence revealed that *A. pisum* fed on *V. faba* could be born with a high level of L-DOPA, and maternally transmitted to offspring during the embryo’s development. This indicates that L-DOPA adaptation system may be well developed at the very beginning of the *A. pisum* life-cycle. We found that most L-DOPA was located in the head in the *A. pisum* fed on *T. repens* (Fig. 2B), and the reason could be that most L-DOPA was synthesized by dopaminergic neurons in its brain without external assimilation. Sexual females and eggs contained more L-DOPA in the *A. pisum* fed on both *V. faba* and *T. repens*, indicating that L-DOPA might have specific functions in the eggs. Based on the tanning phenomenon in eggs, the high amount of L-DOPA in eggs could be used in melanization for absorbing heat and sclerotization, and for preventing the attacks from natural enemies. These results were similar to Eisner’s work between pyrrolizidine alkaloids in leguminous plants and the moth *U. ornatrix*, which has been reported that some plant defense chemicals are concentrated and transferred from mothers to eggs for egg-protection^11^,^12^,^13^. However, more researches are needed to confirm the function of L-DOPA in aphid eggs.

After using an artificial diet to disturb the assimilation of L-DOPA in *A. pisum*, the biosynthesis of L-DOPA was enhanced by up-regulation of related gene *TH*. These results revealed that the aphids fed on *V. faba* would try to stabilize the amount of L-DOPA while its assimilation disrupted. The concentration of L-DOPA in 5 days after treatment revealed that L-DOPA in *A. pisum* was in a dynamic balance (Fig. 3A), and could decline without continuous accumulating. Compared with expression levels of *TH* of *A. pisum* fed on *V. faba*, *TH* of *A. pisum* fed on *T. repens* was quite different. On the contrary, the transcriptional level of *TH* in the aphid feeding on the artificial diet was down-regulated. We suspect that *A. pisum* in high L-DOPA diet conditions might have L-DOPA stabilize system, and this functions may be lost if the aphids are transferred into low L-DOPA diet conditions after they adapted there.

Melanization and encapsulation in *A. pisum* have been previously reported^6^,^7^,^8^. The dark-wounds healing experiment showed different melanization efficiency with or without L-DOPA, and these results verified the correlation between L-DOPA storage and melanization in the wounds (Fig. 4A). Melanization reactions in *A. pisum* wounds from the two host plants were relatively identical, but we observed that melanization in *A. pisum* fed on *T. repens* was not as dark as that in the aphids from *V. faba* at the very beginning of the experiment, and this phenomenon might be caused by the differences in L-DOPA quantity. Stored L-DOPA could enhance melanization in pea aphid, and on the other hand, *A. pisum* fed on *T. repens* might modify the melanization strategy in order to adapt to low L-DOPA situation. Our results also showed that L-DOPA might be related to wound melanization and healing. In an early study, Laughton^7^ analyzed phenoloxidase (PO) activity in *A. pisum*, and found that neither wounding nor injection of bacteria significantly increased rates of PO activity in aphid haemolymph. In contrast, we did not find any expression difference between the two selected genes *proPO1* and *proPO2* at the transcriptional level (Fig. 4G). The possible explanation might be the absence of peptidoglycan receptor proteins (PGRPs), which activate the PO cascade in other insects following bacterial infection^6^. The mechanisms of melanization phenomenon in *A. pisum* need to be further studied.

Previous researches have already demonstrated that UVA irradiation can cause melanization in insect tissues^38^,^39^,^40^. The UVA resistance is always related to melanization level in many insect species^34^,^35^,^36^,^37^. The functions of the insect L-DOPA-dopamine pathway in melanic pigmentation suggest a connection between this physical (UVA) resistance and chemical (L-DOPA) concentration. Our results showed the dynamic changes of L-DOPA level (Fig. 6A) and related genes (*TH*) in *A. pisum* after UVA- radiation (Fig. 6B). The high level of L-DOPA could benefit *A. pisum* via melanization to enhance the UVA-resistance. We also found that the expressions of the two *proPO* genes decreased slowly during all the process in two hosts. Because the expression of *proPO* was stable, the down-regulation of these two genes could be caused by stressful environmental conditions, and the relatively high *proPO1* level (Fig. 6C) in *A. pisum* from *V. faba* demonstrated that *A. pisum* with higher L-DOPA had relatively better condition to protect them from UVA-irradiation. Because L-DOPA has multiple functions in pigmentation, neurotransmission and immune response^9^,^19^,^20^,^33^, we believed that the high amount of L-DOPA in *A. pisum* could have more advantages in pathogen resistance, wound healing, and immunity to other attacks or damages.

We also found that the L-DOPA assimilation would be enhanced if they had L-DOPA resource when *A. pisum* from *V. faba* was in L-DOPA consumption situation (especially in UVA-irradiation, Table 1). On the contrary, *A. pisum* from *T. repens* did not present such reaction in this situation. Although we had transferred some treated individuals to L-DOPA rich diet (*V. faba*), they did not show any more assimilation in L-DOPA than control (Table 1). We believed that they could synthesize L-DOPA themselves to cope this situation (Table 1). This finding revealed that *A. pisum* from *V. faba* would be flexible in L-DOPA modification but *A. pisum* with the same genetic background from *T. repens* lost it.

## Conclusion

At present study, we found that *A. pisum* fed on a high concentration L-DOPA diet could sequester L-DOPA. As far as we have known, this was the first record in chemical sequestration of toxic, non-protein amino acids in insects. A high amount of L-DOPA in *A. pisum* plays important roles in wound healing and physical resistance to UVA damage. Our finding showed that sequestrated chemical such as L-DOPA could be used in resistance and healing to physical damage, and they even can enhance L-DOPA assimilation when needed. This phenomenon reveals that some herbivores could effectively manage harmful host-derived chemicals and integrate them into their own system for their benefits. Although we have known that L-DOPA sequestration has provided *A. pisum* some advantages in ecological competition, more researches are needed to elicit the mechanisms of L-DOPA sequestration and how L-DOPA storage and throttle system work. In addition, more works are needed to confirm these assumptions that L-DOPA in aphids can be used in parasitic and pathogen resistance.

## Materials and Methods

### Experimental insects and plants

A green strain of the pea aphid, *A. pisum*, was cultured on the broad bean (*Vicia faba* L., var. ‘Jinnong’) under long-day conditions (16L: 8D; 20 ± 1 °C) for more than 30 generations at the Key Laboratory of Applied Entomology, Northwest A&F University, China. The aphids were then reared on the white clover *Trifolium repens* (var. ‘Baisanye’) for more than 30 generations in a climate chamber under long-day conditions (16L: 8D; 20 ± 1 °C) before the experiments. All aphids were reared at low density (less than 30 individuals per plant) for more than three generations before experimented.

Two other aphid species, *M. persicae* and *S. avenae*, were used for comparison in L-DOPA concentration assay and toxicity testing as described in the Supplementary Information Fig. S2.

### Transcription analysis

Experimental aphids were quickly-frozen by liquid nitrogen immediately after collection. RNA was extracted with RNAiso Plus (Takara, Japan). cDNA was synthesized using a PrimeScript^TM^ RT reagent Kit with gDNA Eraser (Takara, Japan). Quantitative real-time PCR (qRT-PCR) was performed with SYBR^®^ Premix Ex Taq^TM^ II (Takara, Japan) in an IQ-5 system (Bio-Rad, USA).

Tyrosine pathway regulation in *A. pisum* embryonic and early nymphal stage have been studied, and most key genes in the phenylalanine, tyrosine and dopamine pathways have been identified^42^. The primers of those genes (*TH* and *proPO* for TH and PO, respectively) were designed by Primer-BLAST of NCBI online (http://www.ncbi.nlm.nih.gov/tools/primer-blast/index.cgi?LINK_LOC=BlastHome) and were shown in Table S2.

### Toxicity of L-DOPA to Aphids

In this experiment, two artificial diets were used to rear aphids: one was a normal diet without L-DOPA based on the recipe of Febvay^43^ (coded as AD), the other one was the same diet but with 10 mM L-DOPA added (coded as AD + L-DOPA). Wingless *A. pisum* adults were collected from *V. faba* and *T. repens,* and were reared on each of the two artificial diets for one week. The same artificial diets and treatments were used for wingless *M. persicae*. In addition, *S. avenae* and *R. padi* were treated on another artificial diet based on the studies of Deng and Zhao^44^. The bioassay was shown in Fig. S3F. The survival rates in different treatments were assessed with five replications.

### L-DOPA and dopamine extraction and assay

Ten grams of aphids were placed in a 1.5 ml centrifuge tube. The aphids were crushed by grinding into homogenate in 1 ml extraction solution (0.05 M HCl in 50% ethyl alcohol). The homogenate was then centrifuged at 20,000 g for 10 min at 4 °C^45^,^46^. Final samples were analyzed by an LTQ XL linear ion trap mass spectrometer (Thermo Scientific, Waltham, MA, USA). L-DOPA and dopamine were scanned and fragmented using data dependent MS/MS. Masses of precursor and product ions for each amino acid were described in Table S1. Full daughter scan MS Spectra and selected ion retention time (min) of dopamine and L-DOPA were shown in Fig. S1. All data were acquired and processed using Xcalibur 2.1 software (Thermo Scientific, Waltham, MA, USA). Quantification was achieved by external standard L-DOPA and dopamine mixture of known concentrations.

#### Concentration assay of L-DOPA in plants

Leaves of *V. faba, T. repens, C. annuum, N. tabacum* and *T. aestivum* were freeze-dried for 36 hours in lyophilizer (MSA3.6P, Sartorius, Germany). The samples were then moved into a drying oven (Thermo, USA) and dried for 2 hours at 60 °C. After that, 10 mg of treated samples were weighed precisely by a high precision electronic balance (LL3000, Thermo, USA) at room temperature and transferred into 50 ml plastic tubes and covered. All organized samples were then used for L-DOPA and dopamine extraction and assay.

To analyze relative L-DOPA concentrations in plants phloem, 100 g of leaves of *V. faba, T. repens, C. annuum, N. tabacum* and *T. aestivum* were cut into 5 pieces and extracted in 1 ml EDTA solution (5 mM, pH = 7.0) for 24 hours (20 ± 1 °C). Extracted solutions were then filtered into a new tube; and 100 mg leaves of *V. faba* were extracted by grinding as a positive control^47^,^48^,^49^. Each treatment was replicated six times.

#### Concentration assay of L-DOPA and dopamine in aphids

Wingless *A. pisum* and *M. persicae* (*V. faba* adapted) reared on *V. faba.* Wingless *A. pisum* on *T. repens, M. persicae* from *C. annuum* and *N. tabacum*, Wingless *S. avenae* and *R. padi* from *T. aestivum* were all collected and were used for comparison. Five replicates were used in each aphid species. The samples were then used for L-DOPA, dopamine extraction, and further bioassay.

#### L-DOPA distribution in different morphs and tissues of *A. pisum*

The aphids from *V. faba* and *T. repens* were reared under short-day and low temperature conditions (8L: 16D; 18 ± 1 °C) to induce sexual adults. After two generations, males, sexual females and eggs were collected and used for L-DOPA extraction and bioassay. Ten individuals of wingless adult pea aphids from *V. faba* were dissected into four parts, head, gut, embryo and the remainder in PBS Buffer (pH = 7.2) under a stereomicroscope (JSZ-6, Jiangnan, Jiangsu Province, China). After the heads, guts and embryos were transferred, the remained body tissues were used with PBS buffer as one sample. All samples were then moved into centrifuge tubes with 0.5 ml extraction solution per tube, and processed with the method as described above, and then analyzed using HPLC/MS system. Each treatment was replicated three times.

#### Disruption of L-DOPA assimilation in *A. pisum* fed on *V. faba*

Third instar wingless *A. pisum* from *V. faba* were reared on the artificial diets (no L-DOPA added). L-DOPA concentration was monitored in 12-h intervals for 5 days. Each treatment had three replicates.

The related gene (*TH*) of L-DOPA synthesis pathway was also monitored. Third instar wingless *A. pisum* and *T. repens* from *V. faba* fed on the artificial diets. Aphid samples were collected 2 days after treatment. Three replications were prepared for RNA extraction and *TH* expression measurements.

### Function Analysis of L-DOPA

#### Analysis of melanization efficiency in wounds

*A. pisum* from *V. faba* and *T. repens* and L-DOPA (20 mM in the artificial diet), α-methyltyrosine (*TH* inhibitor, 20 mM in the artificial diet) treated *A. pisum* (*V. faba*) for 5 days were prepared for abdomen-prick experiments. Each individual was pricked with 2 to 5 wounds depending on their size using a glass needle (P-97 Micropipette Puller, Sutter, CA, USA) in pulling program: Pull = 100, VEL = 100, and Time = 100. Badly-wounded individuals were removed and the remainders were reared on a leaf of the host plant in a plastic petri dish (φ35 mm). The aphids were monitored for 3 hours, images were captured every 30 min by digital camera (Olympus pen E-P5, Olympus Corporation, Tokyo, Japan; Lens: Olympus SZ61 stereomicroscope, Olympus Corporation, Tokyo, Japan), camera parameters were set in Manual (shutter speed: 1/200; aperture: depended on lens; ISO: 200; white balance: color temp, 5600K),. The numbers of wounds were counted every 30 min, and the colors (hue, saturation and brightness) in the wounds were analyzed using Photoshop CS6 (Version 13.0 × 64, Adobe, California, USA)^50^.

The expression level of genes *proPO1* and *proPO2* related to melanization in *A. pisum* were measured 1.5 hours after the aphids had been mechanically damaged. Each treatment had replicated three times, and 10 aphids were treated each time.

#### UVA treatment

After many attempts, 50 third instar wingless *A. pisum* nymphs from *V. faba* and *T. repens* were treated under UVA (365.0 nm, WFH-204B, Shanghai, China) irradiation for 1 h. The treated aphids were then moved to their original hosts for 72 h. The aphids were assessed for body-color, melanin and mortality. L-DOPA concentrations and expression levels of relative genes (*TH, proPO1* and *proPO2*) of the aphids were measured in 10 min-intervals for 72 hours. Thereafter, the parameters were measured for every 12 hours.

Wingless *A. pisum* adults from *V. faba* and *T. repens* were placed under UV-irradiation for 60 min. Each replicate had 30 individuals. After treatment, the aphids were individually reared on a leaf of their original host, which was maintained in a plastic Petri dish (φ35 mm) with 1% agar in the bottom for moisturizing. The old leaves were replaced by new ones every two days. Deformity rates in their descendants were analyzed daily for 10 days. Each treatment was replicated three times.

#### L-DOPA assimilation analysis of *A. pisum* with wounds and UV-irradiation

Four instar wingless nymphs of *A. pisum* from *V. faba* and *T. repens* were collected for experiments. Twenty individuals were used for each treatment and starved for 6 hours before experiments as shown in Table 1. The treated aphids (5 wounds on each aphid for the injury treatment, and 30 min UVA irradiation for the UVA treatment) were transferred into a new host. The samples were then collected for L-DOPA content analysis after 3 hours. Each treatment was replicated three times.

### Statistical analysis

L-DOPA concentrations in plants and aphids were subjected to analysis of a variance (ANOVA); means were separated using Duncan test at *P* < 0.05. All other experimental data were analyzed using Student’s *t*-test, or Chi-square test (version 22; SPSS Inc., Chicago, IL, USA).

## Additional Information

**How to cite this article**: Zhang, Y. *et al.* Pea aphid *Acyrthosiphon pisum* sequesters plant-derived secondary metabolite L-DOPA for wound healing and UVA resistance. *Sci. Rep.* **6**, 23618; doi: 10.1038/srep23618 (2016).

## Supplementary Material

Supplementary Information

## Figures and Tables

**Table 1:** L-DOPA contents analysis of *Acyrthosiphon pisum* in the injured, UVA-treated and untreated aphids after they were transferred from their original host plant (*Vicia faba or Trifolium repens*) to a different host plant (*V. faba or T. repens*).

Contents of L-DOPA, mean (ng/mg) ± SE^x^								
Original host	*Vicia faba*				*Trifolium repens*			
Transferred host	*Vicia faba*	*Trifolium repens*	*T*	*P*	*Vicia faba*	*Trifolium repens*	*T*	*P*
UVA treated (365 nm)	78.988 ± 6.798a	45.949 ± 5.971a	3.651	0.022	4.5789 ± 2.093a	4.6698 ± 1.982a	−0.032	0.976
Injured	65.1110 ± 7.828ab	60.5256 ± 11.063a	0.321	0.758	3.8763 ± 0.103a	3.5569 ± 0.590a	1.123	0.299
Control	51.0561 ± 2.324b	59.9765 ± 8.943a	−0.965	0.363	4.0234 ± 1.300a	3.1998 ± 0.857a	0.529	0.611
*F*_*2,10*_	6.011	0.555			0.080	0.475		
*P*	0.022	0.591			0.923	0.636		

^x^Means in the same column among the three treatments and between the two host plants in the same sub-column with the same letters are not significantly different (*P* < 0.05, Duncan test); and the data in each sub-row are analyzed by Student’s *t*-test.
