# Supplementary material for: Pea aphid Acyrthosiphon pisum sequesters plant-derived secondary metabolite L-DOPA for wound healing and UVA resistance
Source: Sci Rep. 2016 Mar 23;6:23618. doi: 10.1038/srep23618 (PMC4804291; doi:10.1038/srep23618)
Supplement: Supplementary Information [file srep23618-s1.pdf]

**Supplementary Information**

# Pea aphid *Acyrthosiphon pisum* sequesters plant-derived secondary metabolite L-DOPA for wound healing and UV resistance

Yi Zhang, Xing-Xing Wang, Zhan-Feng Zhang, Nan Chen, Jing-Yun Zhu, Hong-Gang Tian, Yong-Liang Fan, Tong-Xian Liu[x]

Table. S1. Primers used for quantitative PCR

| Genes | ACYPI Identifier (or GenBank) | Gene Name | Forward primer | Reverse primer |
|---|---|---|---|---|
| *TH* | ACYPI008168 | Tyrosine Hydroxylase EC 1.14.16.2 | ATACATACCGTTTCCGTCTG | TCGTCTAGTTTCGTTTGTGT |
| *proPO1* | ACYPI001367 | Prophenoloxidase 1 EC 1.14.18.1 | ATTCAGGGAACAGAGGAAAC | GCTGCGTCTGAATTATTAGC |
| *proPO2* | ACYPI004484 | Prophenoloxidase 2 EC 1.14.18.1 | CCGTAGCATTGATACACAGA | ATTTCGATGGGAGTCCTAGA |

Table S2. Masses of precursor and product ions and collision energy for liquid chromatography-electrospray ionization tandem mass spectrometry (LC-ESI-MS-MS) analysis of L-DOPA and dopamine

| Compounds | Precursor ion $(M+H)^{+}$(*m/z*) | Product ion (*m/z*) | Collision energy (eV) |
|---|---|---|---|
| L-DOPA | 198 | 152 | 35 |
| Dopamine | 154 | 137 | 35 |

Fig. S1. Full daughter scan MS Spectra and selected ion retention time (min) of dopamine (A, B and C) and L-DOPA (D and E). MS Spectra information was referred to the database of METLIN (Scripps Center for Metabolomics METLIN: Metabolite and Tandem MS Database http://metlin.scripps.edu/terms.php). Liquid chromatography separations were carried out with XTerra MS C18 Column (125Å pore size, 5 μm, 150 mm ×4.6 mm; Waters Corp., Milford, MA, USA). Elution was performed by applying a three-step gradient: 100% A for 8 min, 0-100% B linear for 2 min, 100% B for another 5 min, and 0–100% A linear for 1 min, holding the system at 100% A for 8 min. Mobile phase A was an aqueous solution containing 15% methanol and 0.1% formic acid; and mobile phase B was 100% methanol with 0.1% formic acid. The flow rate was 0.6 mL/min. The mass spectrometer was set in the positive electrospray ionization mode. Nitrogen was used as the sheath gas (40 arbitrary units) and auxiliary gas (10 arbitrary units). The spray voltage was set at 4.5 kV and the ion transfer capillary temperature was 275 ℃.

Fig. S2. L-DOPA contents difference in *V. faba*, *T. repens*, *C. annuum*, *N. tabacum* and *T. aestivum*. (A), and L-DOPA, dopamine contents difference in *A. pisum*, *M. persicae* (*V. faba*, *C. annuum* and *N. tabacum*), *S. avenae* and *S. graminum* (B, C), Two other aphid species were used for L-DOPA concentration assay and toxicity testing: the green peach aphid, *Myzus persicae* Sulzer, which were originally collected from bell pepper, *Capsicum annuum* L. (var. 'JingYuanXinWangZi') and tobacco *Nicotiana tabacum* L. (var. 'Qinyan 95') from Yangling, Shaanxi, China, The English grain aphid, *S. avenae*, and the bird cherry-oat aphid, *Rhopalosiphum padi* L., which were collected from wheat, *Triticum aestivum* (var. 'Xinong 979') from Yangling, Shaanxi, China. All aphids were cultured at the same environmental conditions (16L: 8D; 20±1 ℃) as for A. pisum as previously described. Each value represents the mean±SEM. Different letters above bars indicate statistically significant differences ($P<0.05$, Duncan test). L-DOPA concentration in phloem of hosts were detected and showed in D, L-DOPA amounts in leaves of *V. faba* were extracted by grinding as a positive control, Each value represents the mean±SEM from independent determinations (* $P<0.05$, ** $P<0.01$, Student's *t*-test).

Fig. S3. Survival rate of all experimental aphids treated with L-DOPA (20mM) by feeding artificial diets. *M. persicae* (*V. faba*) survival rate ($n_{AD}$=120, $n_{DOPA}$=120) was shown in (A); *M. persicae* (*C. annuum*) survival rate ($n_{AD}$ =116, $n_{DOPA}$=100) was shown in (B); *M. persicae* (*N. tabacum*) survival rate ($n_{AD}$ =80, $n_{DOPA}$=100) was shown in (C); *R. padi* (*T. aestivum*) survival rate ($n_{AD}$=106, $n_{DOPA}$=108) was shown in (D); *S. avenae* (*T. aestivum*) survival rate ($n_{AD}$=106, $n_{DOPA}$=108) was shown in (E). the device for rearing was shown in (F)

Fig. S1

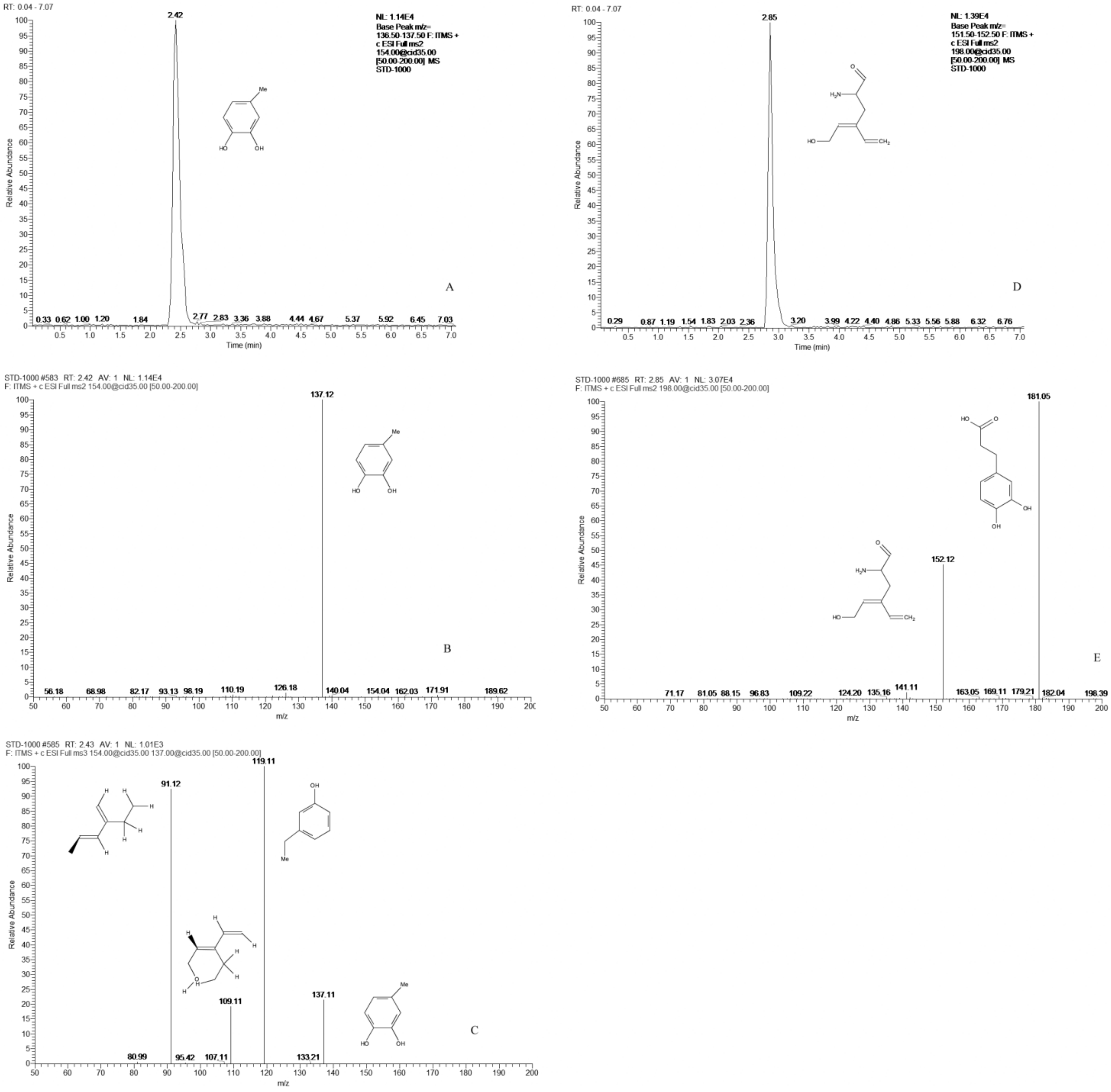

RT: 0.04 - 7.07
NL: 1.14E4
Base Peak m/z= 136.50-137.50 F: ITMS + c ESI Full ms2 154.00@cid35.00 [50.00-200.00] MS STD-1000
Relative Abundance
Time (min)
A
RT: 0.04 - 7.07
NL: 1.39E4
Base Peak m/z= 151.50-152.50 F: ITMS + c ESI Full ms2 198.00@cid35.00 [50.00-200.00] MS STD-1000
D
STD-1000 #583 RT: 2.42 AV: 1 NL: 1.14E4
F: ITMS + c ESI Full ms2 154.00@cid35.00 [50.00-200.00]
m/z
B
STD-1000 #685 RT: 2.85 AV: 1 NL: 3.07E4
F: ITMS + c ESI Full ms2 198.00@cid35.00 [50.00-200.00]
E
STD-1000 #585 RT: 2.43 AV: 1 NL: 1.01E3
F: ITMS + c ESI Full ms3 154.00@cid35.00 137.00@cid35.00 [50.00-200.00]
C

Fig. S2

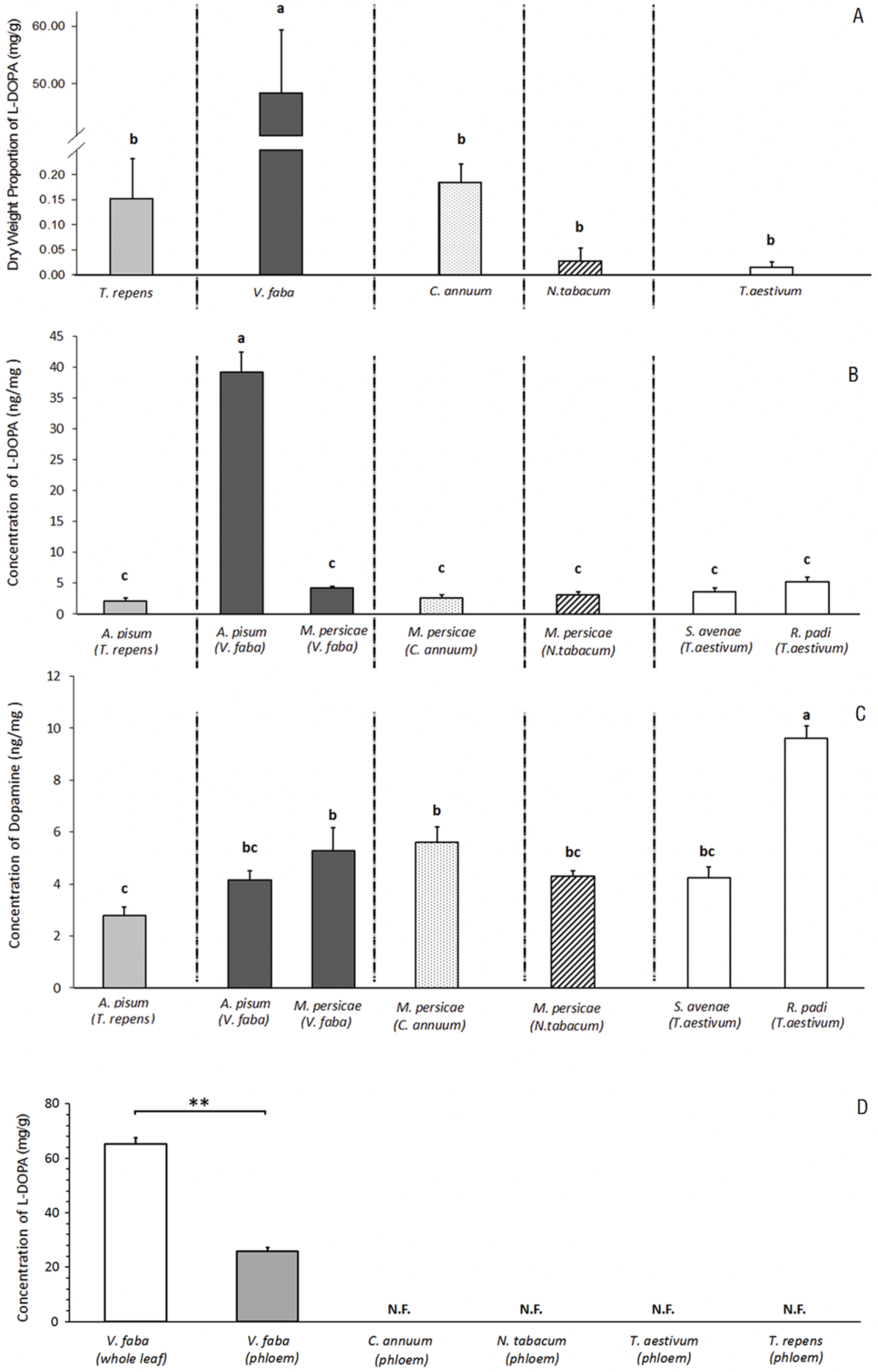
A
Dry Weight Proportion of L-DOPA (mg/g)
60.00
50.00
0.20
0.15
0.10
0.05
0.00
a
b
T. repens
V. faba
C. annuum
N.tabacum
T.aestivum
B
Concentration of L-DOPA (ng/mg )
45
40
35
30
25
20
15
10
5
0
A. pisum (T. repens)
A. pisum (V. faba)
M. persicae (V. faba)
M. persicae (C. annuum)
M. persicae (N.tabacum)
S. avenae (T.aestivum)
R. padi (T.aestivum)
C
Concentration of Dopamine (ng/mg )
12
10
8
6
4
2
0
a
b
bc
c
D
Concentration of L-DOPA (mg/g)
80
60
40
20
0
**
N.F.
V. faba (whole leaf)
V. faba (phloem)
C. annuum (phloem)
N. tabacum (phloem)
T. aestivum (phloem)
T. repens (phloem)

Fig. S3

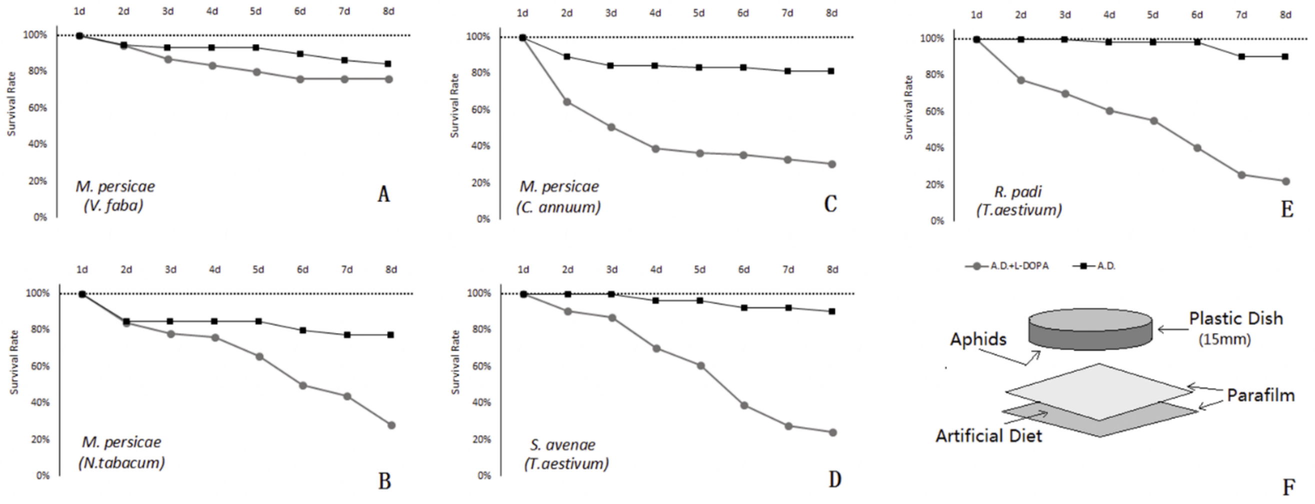

M. persicae
(V. faba)
A
M. persicae
(C. annuum)
C
R. padi
(T.aestivum)
E
M. persicae
(N.tabacum)
B
S. avenae
(T.aestivum)
D
Survival Rate
A.D.+L-DOPA
A.D.
Plastic Dish
(15mm)
Aphids
Parafilm
Artificial Diet
F


67

68
